# An Environmentally Friendly Superhydrophobic Wood Sponge with Photo/Electrothermal Effects Prepared from Natural Wood for All-Weather High-Viscosity Oil–Water Separation

**DOI:** 10.3390/polym16233256

**Published:** 2024-11-23

**Authors:** Kenan Yang, Sainan Wang, Bin Du, Shisheng Zhou

**Affiliations:** 1 School of Mechanical and Precision Instrument Engineering, Xi’an University of Technology, Xi’an 710054, China; 1200210002@stu.xaut.edu.cn; 2 Shaanxi Collaborative Innovation Center of Green Intelligent Printing and Packaging, Xi’an University of Technology, Xi’an 710054, China; 18110737432@163.com (S.W.); dubin@xaut.edu.cn (B.D.); 3 Faculty of Printing, Packaging Engineering and Digital Media Technology, Xi’an University of Technology, Xi’an 710054, China

**Keywords:** photothermal, joule heating, high-viscosity oil–water separation, superhydrophobic wood sponge

## Abstract

Rapid industrial development has led to increased crude oil extraction and oily wastewater discharge. Achieving oil–water separation and marine oil adsorption in a cost-effective, efficient, and environmentally friendly manner remains a global challenge. In this work, natural wood was chemically treated to prepare a degradable and environmentally friendly wood sponge structure. In situ polymerization and spraying methods were used to produce an environmentally friendly oil–water separation sponge with superhydrophobic and superoleophilic properties (Fe_3_O_4_@P-P@WS). Fe_3_O_4_@P-P@WS had excellent superhydrophobicity (WCA = 154.2°) and self-cleaning properties. Additionally, Fe_3_O_4_@P-P@WS could convert solar and electrical energy into thermal energy, reaching a surface temperature of 74 °C under sunlight irradiation with an intensity of 1.0 kW m^−2^. When a voltage of 9 V was applied, the surface temperature reached 120.5 °C. Moreover, under the suction of a vacuum pump or the action of gravity, the continuous separation of highly fluid oil substances was achieved. The designed Fe_3_O_4_@P-P@WS offers advantages such as easily obtained raw materials, energy efficiency, simple preparation, and the ability to solve secondary pollution issues, providing a new technology for cleaning organic matter in industrial wastewater discharge and for round-the-clock cleaning of high-viscosity crude oil leaked during offshore oil exploitation.

## 1. Introduction

A growing population and rapid industrialization have significantly increased the discharge of organic pollutants into aquatic environments, contributing to severe water contamination and ecological degradation [1,2,3]. To alleviate the detrimental impact of oily wastewater on ecosystems and human health, the development of efficient and practical oil–water separation technologies is of paramount importance. Recently, investigations into fascinating natural phenomena such as the collection of morning dew on lotus leaves and the effortless movement of water striders on water have inspired the discovery of superhydrophobic surfaces. These surfaces exhibit remarkable water-repellent properties, characterized by a water contact angle (WCA) exceeding 150° and a sliding angle of less than 10° [4,5,6]. The combined hydrophobicity and oleophilicity of these surfaces make them highly suitable for applications in oil–water separation.

There are two primary approaches to constructing superhydrophobic surfaces: coating the surface with low-surface-energy materials and creating micro/nano-scale roughness on the surface [7,8]. Nanoparticles have become a preferred option for constructing micro/nano structures. However, nanoparticles exhibit high surface energy and are therefore in a thermodynamically unstable state, are prone to agglomeration, and are difficult to mix with organic solvents, leading to the uneven dispersion of nanoparticles in such solvents. Surface modification can reduce the surface polarity of nanoparticles, improving their dispersion performance in organic solvents [9,10].

Currently, the superhydrophobic materials utilized in oil–water separation include two-dimensional superhydrophobic films and papers [11,12,13,14], and three-dimensional superhydrophobic sponges and aerogels have become research hotspots in recent years [15,16,17,18]. Most superhydrophobic sponges are based on polyurethane sponges, melamine sponges, and similar materials [19,20]. However, these sponges may cause secondary pollution during production or recycling. In 2018, Hu et al. [21] created an anisotropic delignified wood sponge by employing chemical treatment to selectively remove lignin from natural basswood. Preparing sponge substrates from natural, renewable wood effectively addresses the issue of secondary pollution.

The composition of wood can be mainly divided into two categories. One is the phenolic compound lignin, and the other comprises the polysaccharide carbohydrates cellulose and hemicellulose. Moreover, wood contains a unique internal pore structure [22,23,24]. These pores facilitate the penetration of solutions into the wood, allowing for enhanced delignification and hemicellulose removal. A growing body of research has focused on superhydrophobic materials based on wood sponges [25,26,27,28].

It is worth noting that during oil–water separation experiments, it is difficult for sponges to absorb some high-viscosity oils at room temperature. It has been reported that the viscosity of oils is significantly affected by temperature; as the temperature rises, the viscosity decreases [29]. Therefore, modifying the substrate with materials such as polydopamine (PDA) and Fe_3_O_4_, which possess excellent photothermal properties, can produce superhydrophobic sponges with in situ self-heating capabilities, providing a solution for high-viscosity oil–water separation [30,31,32,33,34]. However, at present, there is a relative lack of research on the preparation of superhydrophobic sponges with self-heating properties by using wood sponges as substrates. Meanwhile, sunlight is limited throughout the day, and the solar intensity varies at different times and locations. Even at the same time and place, weather conditions may result in insufficient sunlight, affecting absorption efficiency. Therefore, in this study, for the first time, natural and degradable wood sponges were used as substrates. Through modifying them, a superhydrophobic wood sponge that can convert both light energy into thermal energy and electrical energy into thermal energy was developed, which can achieve the adsorption of heavy oil all day long.

In this study, a degradable three-dimensional oil–water separation material suitable for all-weather oil adsorption was prepared. Balsa wood was selected as the source of raw materials for this experiment because of its advantages, such as its light weight and high porosity. Lignin and hemicellulose were selectively removed through chemical treatment. After freeze-drying, a wood sponge (WS) skeleton composed of cellulose was obtained. To further achieve self-heating and superhydrophobic properties, PDA was deposited onto the surface of the WS using in situ polymerization, resulting in a PDA-modified WS (PDA@WS). The surface of the PDA@WS was then coated with polypyrrole (PPy) through in situ polymerization to produce P-P@WS with excellent electrical conductivity. After further surface modification with octadecyltrimethoxysilane (C18TMS), Fe_3_O_4_ with good dispersion performance in organic solvents was sprayed onto the P-P@WS along with polydimethylsiloxane (PDMS), ultimately producing a superhydrophobic WS (Fe_3_O_4_@P-P@WS).

## 2. Materials and Methods

### 2.1. Materials

Polydimethylsiloxane (PDMS), methylene blue (MB), pyrrole (Py), C18TMS (90%), nano-Fe_3_O_4_ (Fe_3_O_4_ nanoparticles, 20 nm), Oil Red O, and sodium hydroxide (NaOH, 96%) were procured from Henan Aierfeng Chemical Co., Ltd. (Jiaozuo, China). Sodium chlorite (NaClO_2_, 80%), ferric chloride hexahydrate (FeCl_3_·6H_2_O), glacial acetic acid (CH_3_COOH), sodium dodecylbenzene sulfonate (SDBS), and ammonia solution (NH_3_·H_2_O, 25%) were sourced from Tianjin Xinjinhua Chemical Co., Ltd. (Tianjin, China). Additionally, n-hexane, 1,2-dichloroethane, anhydrous ethanol, toluene, acetone, and dopamine hydrochloride (DA, 98%) were acquired from Fuyu Fine Chemical Co., Ltd. (Tianjin, China). Commercial products such as vegetable oil, pump oil, lard, and balsa wood (100 mg cm^−3^, dimensions 1 cm^3^) were obtained from local suppliers. All chemicals were utilized as received, without further purification.

### 2.2. Preparation of WS

In this experiment, WS was synthesized through a two-step chemical process. First, natural wood (NW) was cut into small cubes, each with dimensions of 1 cm^3^, using a table saw. A 200 g solution of NaClO_2_ at a specific concentration was prepared, and its pH was adjusted to 4.0 using CH_3_COOH. The NW cubes were immersed in this solution and gently stirred at 100 °C for 10 h. After the reaction, the cubes were soaked in deionized water for 12 h to produce delignified wood (DW). Next, a 200 g solution of 7 wt% NaOH was prepared, into which the DW cubes were immersed and reacted at 80 °C for 10 h. Upon completion of the reaction, the cubes were thoroughly rinsed with deionized water until the pH reached neutral (pH = 7). Finally, the washed cubes were frozen at −20 °C for 12 h and subjected to freeze-drying for 30 h, yielding the WS material.

### 2.3. Preparation of PDA@WS

A 30 mL anhydrous ethanol solution was prepared and adjusted to a pH of 8.5 using ammonia solution. Subsequently, 60 mg of DA was added, and the mixture was stirred in the mildly alkaline ethanol solution until complete dissolution was achieved. The WS was then immersed in this solution under vacuum conditions for 30 min. The reaction was allowed to proceed at room temperature and atmospheric pressure for 12 h. Following this, the WS was removed and dried in a vacuum oven at 60 °C for 3 h, yielding PDA@WS.

### 2.4. Preparation of P-P@WS

Twenty milligrams of SDBS and 415 µL of Py were dissolved in 50 mL of deionized water, resulting in Solution A. The PDA@WS was subsequently immersed in this solution. In a separate procedure, 4.03 g of ferric chloride hexahydrate (FeCl_3_·6H_2_O) was dissolved in 50 mL of deionized water to create Solution B, which was then gradually introduced into Solution A. Following a reaction period of 2 h at 5 °C, the solution was placed in a vacuum environment for 30 min to facilitate vacuum impregnation. The sponge was then removed, frozen at −20 °C for 12 h, and freeze-dried for 24 h, resulting in the formation of P-P@WS.

### 2.5. Preparation of Fe_3_O_4_@P-P@WS

A solution of 90 mL anhydrous ethanol, 10 mL deionized water, and 4 mL of ammonia solution was thoroughly mixed, after which 1.55 g of Fe_3_O_4_ was introduced and the mixture was mechanically stirred for 30 min. Subsequently, 1.25 mL of C18TMS was gradually added, allowing the reaction to proceed at room temperature for 5 h. The resulting product was magnetically separated and repeatedly washed with anhydrous ethanol until neutral pH was achieved. Finally, the product was dried in a vacuum oven at 70 °C for 12 h, ground into a fine powder, and stored for future use.

A total of 1 g PDMS was added to 20 g toluene and stirred until completely dissolved. Then a certain amount of hydrophobically modified Fe_3_O_4_ nanoparticles were added and dispersed uniformly using ultrasonication. A spray gun was used to spray the above dispersion liquid evenly on the surface of P-P@WS multiple times. After drying, Fe_3_O_4_@P-P@WS was obtained.

### 2.6. Sample Characterization and Methods

Scanning electron microscopy (SEM) was carried out using a ZEISS Gemini SEM (Oberkochen, Germany) to examine the microstructural features of the samples. The static water contact angle (WCA) and oil contact angle (OCA) of the sample were measured using a JY-PHb contact angle goniometer (Chende, China). Fourier-transform infrared spectroscopy (FTIR) was performed with a Shimadzu IRSpirit (Kyoto, Japan) to analyze the functional group changes during the preparation process of WS and the surface superhydrophobic modification process. To investigate the self-heating behavior of the sponge under both light and electrical stimulation, a SAN-EI XES-40S3 solar simulator (Tokyo, Japan), RIGOL DP832 (Suzhou, China) programmable DC power supply, and HIKMICRO (Hangzhou, China) handheld thermal imaging camera were employed. The mechanical properties of the samples were evaluated using an Instron 5500 universal testing machine (Boston, MA, USA).

### 2.7. Oil–Water Separation Experiment

In this experiment, various separation devices were designed based on the physical properties of different oils. To visually monitor the oil–water separation process, water was dyed blue with methylene blue (MB), while oil was colored red using Oil Red O. The oils and water were mixed through simple physical agitation. Using an external vacuum pump, oil substances with lower density and viscosity than water were continuously separated from the water’s surface. Fe_3_O_4_@P-P@WS was employed as an intermediate filtration layer to facilitate the continuous separation of low-viscosity, high-density oils through gravity. For the separation of high-viscosity oils, absorption was achieved either under simulated sunlight generated by a solar simulator or via power from a DC power supply. The efficiency of oil–water separation was calculated using the following formula:

(1) $$\eta =\frac{V_{1}}{V_{0}}\times 100\%$$

Here, *V*_1_ stands for the quantity of oil gathered after a single continuous oil–water separation, while *V*_0_ denotes the original amount of oil prior to oil–water separation.

## 3. Results

### 3.1. Preparation and Characterization Regarding of NW, DW, and WS

**Scheme 1:**
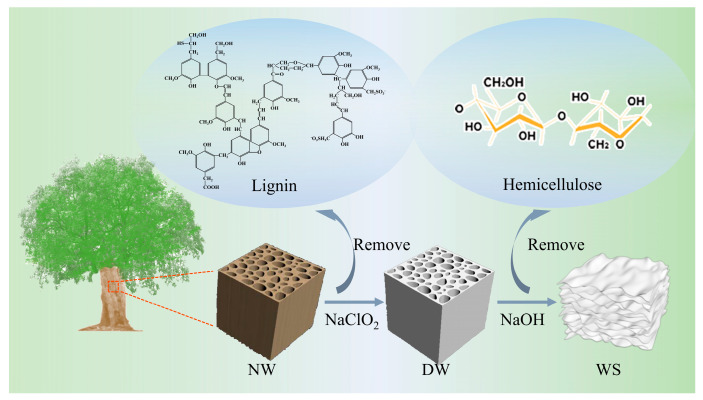
Flowchart of WS preparation process. WS: wood sponge; NW: natural wood; DW: delignified wood.

As illustrated in Scheme 1, the preparation of the sponge skeleton (WS) followed a two-step chemical process. Firstly, natural wood (NW) was treated with NaClO_2_ to selectively remove lignin, yielding delignified wood (DW). Secondly, the DW underwent further treatment with sodium hydroxide (NaOH) to remove hemicellulose. The resulting material was then freeze-dried to form a porous sponge structure composed primarily of cellulose.

**Figure 1:**
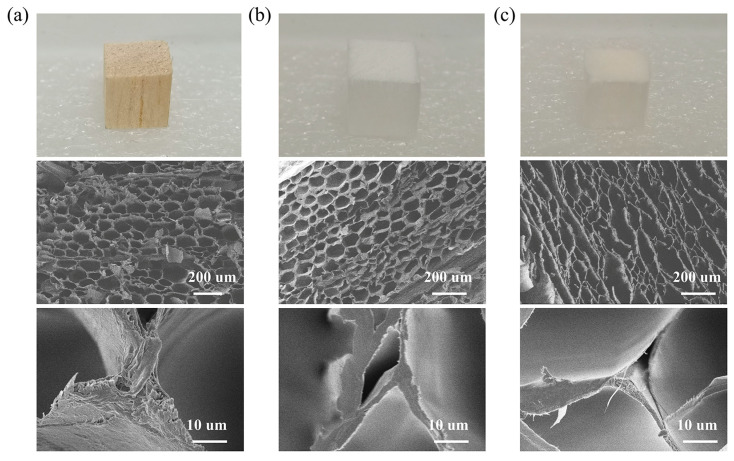
Scanning electron microscope images of (**a**) natural wood (NW), (**b**) delignified wood (DW), and (**c**) wood sponge (WS).

SEM was conducted to characterize and analyze the morphology of NW, DW, and WS. NW appeared as the light yellow color of the wood itself; the cross-section revealed honeycomb-shaped vessel pores for water and nutrient supply, and the cell walls were tightly connected without gaps (Figure 1a). As shown in Figure 1b, following NaClO_2_ treatment, the color of the sponge changed from light yellow to white due to the removal of the dark lignin. At this stage, the sponge skeleton was composed of colorless polysaccharides. The delignified sponge largely retained its porous structure, although some regions exhibited delamination, and small pores appeared between the cell walls.

**Figure 2:**
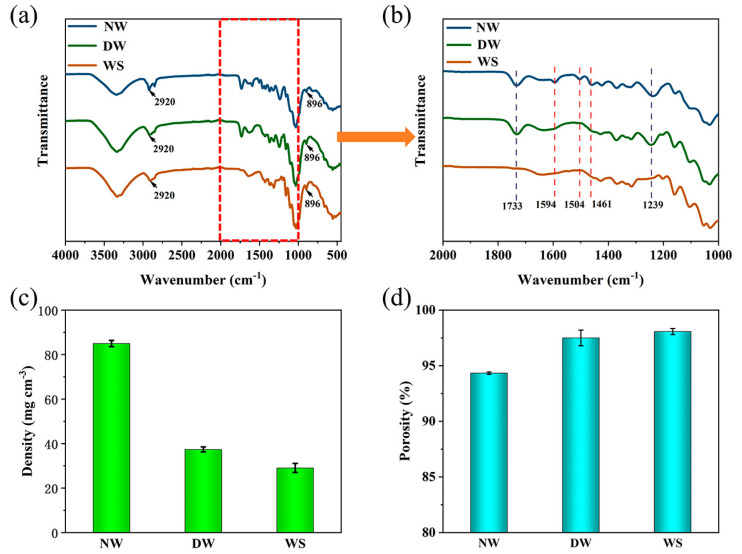
(**a**,**b**) Fourier-transform infrared spectroscopy (FTIR) spectra of NW, DW, and WS. (**c**) Density of NW, DW, and WS. (**d**) Porosity of NW, DW, and WS. NW: natural wood; DW: delignified wood; WS: wood sponge.

FTIR analysis (Figure 2a,b) confirmed that the NaClO_2_ treatment did not affect the peaks at 2920 and 896 cm^−1^, which represented cellulose [35,36]. However, the peaks representing the aromatic skeleton vibrations of lignin at 1594, 1504, and 1461 cm^−1^ disappeared [36,37]. The characteristic peaks at 1733 cm^−1^, corresponding to the stretching vibration of the C=O bond in the acetyl group of hemicellulose, and at 1239 cm^−1^, associated with the stretching vibration of the C=O–C bond in the acetyl group of hemicellulose, are both distinctly preserved [36,38], indicating that NaClO_2_ treatment eliminated lignin while preserving cellulose and hemicellulose. According to the density calculation formula ρ=m/v and porosity calculation formula $p=\left(1-\rho /\rho_{s}\right)\times 100\%$ (where the value of $\rho_{s}$ is the actual density of cellulose 1.5 g cm^−3^), the removal of lignin reduced the sponge’s density from 84.9 ± 1.35 mg cm^−3^ to 37.32 ± 1.2 mg cm^−3^, while the porosity increased from 94.34% ± 0.11 to 97.6% ± 0.7 (Figure 2c,d).

As shown in Figure 1c, following further NaOH treatment, the WS no longer presented a porous structure; rather, it showed a stacked, layered structure with different degrees of cell wall contraction [39,40]. The pore size between the cell walls increased as well. Owing to its layered structure, the WS exhibited the ability to compress under applied stress, effectively storing mechanical energy. Upon the removal of the external force, this stored energy was released, causing the compressed layers to rebound and form arches, thereby displaying remarkable resilience [41]. This excellent elasticity is one of the main reasons why WS can be utilized in the field of three-dimensional oil absorption. Although the FTIR analysis of the WS (Figure 2a,b) still revealed peaks representing cellulose at 2920 and 896 cm^−1^, the peaks representing hemicellulose at 1733 and 1239 cm^−1^ disappeared, confirming the successful removal of hemicellulose. The removal of hemicellulose further reduced the density of the WS (28.88 ± 2.0 mg cm^−3^) and increased its porosity (98.10% ± 0.25) (Figure 2c,d).

**Figure 3:**
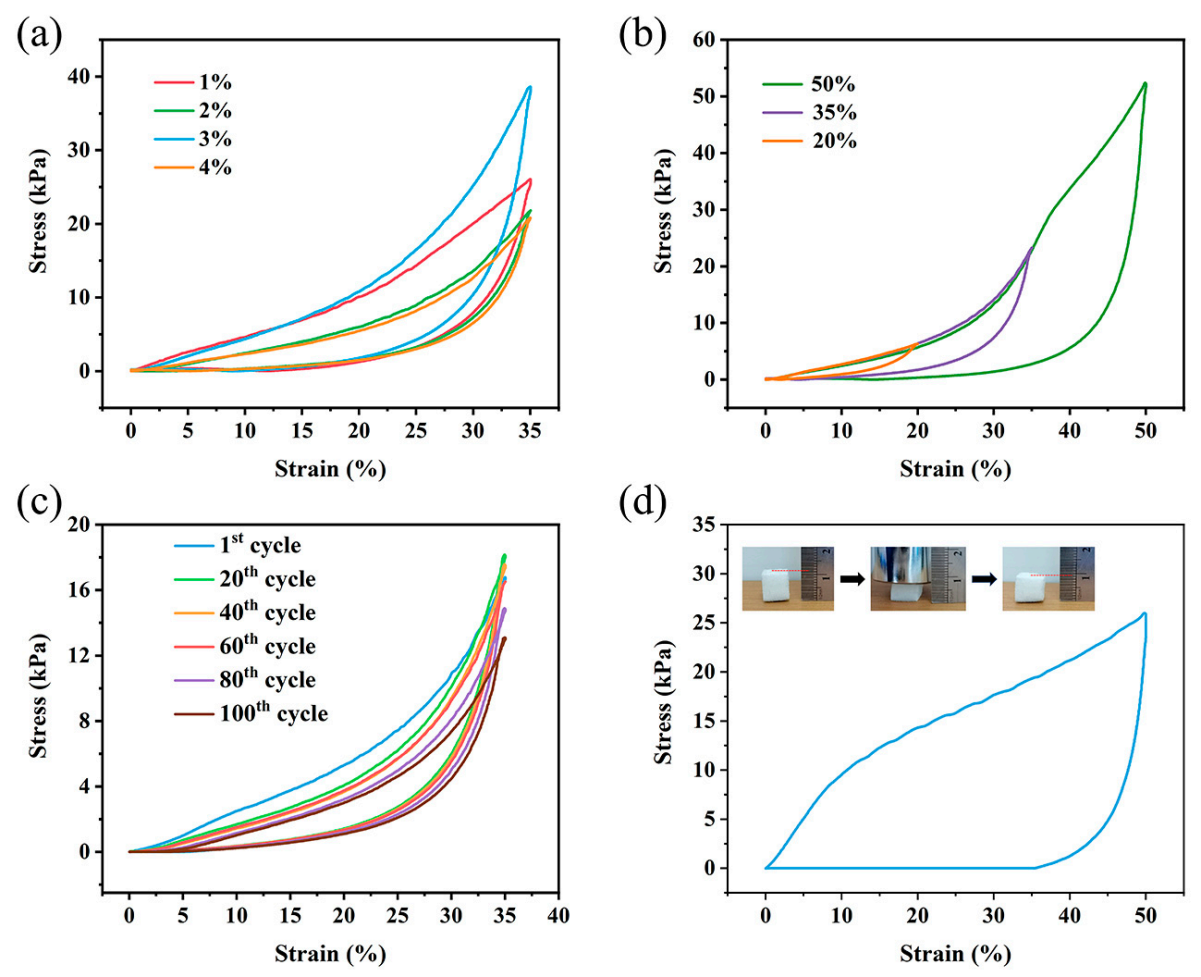
(**a**) Stress–strain curves of WS samples fabricated with varying NaClO_2_ concentrations. (**b**) Stress–strain curves of WS prepared with 2 wt% NaClO_2_ under compressive strains of 20%, 35%, and 50%. (**c**) Compression cycling test of WS at 35% strain for 100 cycles. (**d**) Stress–strain curve of DW under 50% compressive strain. WS: wood sponge; DW: delignified wood.

The compression–rebound characteristics of the WS were characterized by means of a universal testing machine. First, the effects of different NaClO_2_ concentrations on the compressive properties of WS were examined. As shown in Figure 3a, NaClO_2_ concentrations from 1 wt% to 4 wt% were tested with a compression–rebound experiment at 35% strain. When the concentrations of NaClO_2_ were 1 wt% and 4 wt%, the stress–strain curves showed obvious losses in height, indicating that the sponges prepared at these two concentrations underwent plastic deformation under 35% strain. The WS prepared at these two concentrations did not possess a high elastic state. Compared with the concentration of 2 wt%, the area of the hysteresis loop of the stress–strain curve at the concentration of 3 wt% was larger, which demonstrated that more energy was dissipated during the compression process of the wood sponge prepared at this concentration. After comprehensive consideration, this study chose the concentration of 2 wt% for NaClO_2_ to prepare the sponge. Figure 3b displays the stress–strain curves of WS at different compression rates (20%, 35%, and 50%). Due to the elastic deformation of WS, the linear area of the curve at 20% compression was smaller than those at 35% and 50%. After the release of pressure, all three compression rates returned to their original shapes, demonstrating the high elasticity of WS. During compression, structural damage led to energy dissipation, causing the stress–strain curves to exhibit significant hysteresis loops [41].

The WS was subjected to repeated compression–rebound experiments at 35% compression for 100 cycles (Figure 3c). After 100 cycles, the WS still exhibited excellent elasticity, with minimal rebound rate loss, maintaining over 95% height retention. Additionally, the hysteresis loop area of the stress–strain curves gradually decreased, indicating reduced structural damage and energy dissipation within the sponge. The SEM images (Figure 1b) revealed that the DW contained a small amount of layered structure, suggesting some compressibility. Figure 3d shows the DW compression experiment at 50% strain, which indicated that after the release of external force, the DW could not return to its pre-compression height, with a height loss of approximately 35%.

### 3.2. Hydrophobic Modification of Fe_3_O_4_

Fe_3_O_4_ nanoparticles [42] agglomerate easily in solvents due to their small particle size and magnetic properties. This dispersion problem can be addressed by surface hydrophobic modification. In this study, C18TMS, which has long chains, was employed to modify the surface of Fe_3_O_4_ nanoparticles through hydrolysis under alkaline conditions.

**Figure 4:**
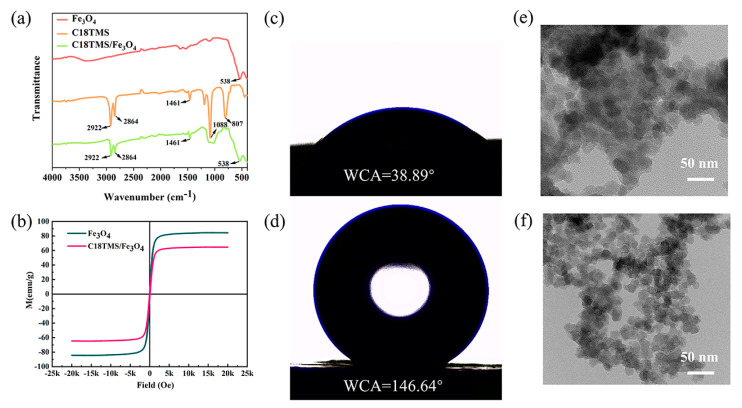
(**a**) FTIR spectra of Fe_3_O_4_, C18TMS, and C18TMS/Fe_3_O_4_. (**b**) Hysteresis loops of Fe_3_O_4_ and C18TMS/Fe_3_O_4_. WCA on (**c**) Fe_3_O_4_ and (**d**) C18TMS/Fe_3_O_4_. TEM images of (**e**) Fe_3_O_4_ and (**f**) C18TMS/Fe_3_O_4_. C18TMS: octadecyltrimethoxysilane; WCA: water contact angle; TEM: transmission electron microscopy.

FTIR analysis (Figure 4a) revealed the presence of characteristic peaks at 2922 cm^−1^ and 2846 cm^−1^, corresponding to the stretching vibrations of the methyl and methylene groups in C18TMS, respectively. Additionally, the peak at 1461 cm^−1^, associated with the C-O bond, appeared in the spectrum of the modified Fe_3_O_4_, although with some reduction in intensity. The characteristic peaks at 1088 cm^−1^ and 807 cm^−1^ in the C18TMS spectrum, attributed to the symmetric and asymmetric stretching vibrations of Si-O-Si bonds, were observed within the 928–1168 cm^−1^ range in the Fe_3_O_4_ spectrum as Si–O and Si-O-Fe vibrations [43,44], confirming the successful hydrophobic modification of Fe_3_O_4_.

Moreover, as shown in Figure 4b, a vibrating sample magnetometer was utilized to measure the Fe_3_O_4_ before and after hydrophobic modification. The results indicated that the nanoparticles exhibited superparamagnetic behavior both before and after modification, with no remanence and only a slight decrease in saturation magnetization following modification. The WCA of Fe_3_O_4_ was measured before and after modification by pressing the nanoparticles into tablets (Figure 4c,d). The WCA increased from a hydrophilic state of 38.89° to a hydrophobic state of 146.64°, confirming the successful hydrophobic modification of Fe_3_O_4_. To further demonstrate the improved dispersibility of the modified particles in organic solvents, transmission electron microscopy (TEM) was conducted to characterize their dispersion in absolute ethanol (Figure 4e,f). The images clearly revealed significant improvement in the dispersion of the particles after modification.

### 3.3. Preparation and Wettability Performance of Fe_3_O_4_@P-P@WS

**Scheme 2:**
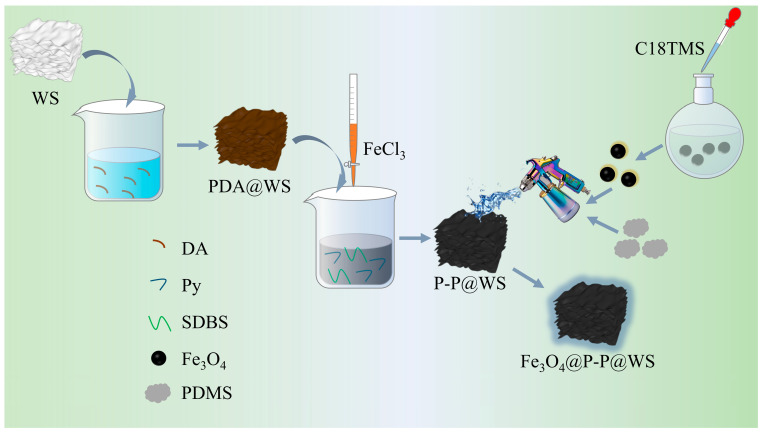
Flowchart of Fe_3_O_4_@P-P@WS preparation process. WS: wood sponge; PDA@WS: polydopamine-modified wood sponge; P-P@WS: polypyrrole-coated polydopamine-modified wood sponge; Fe_3_O_4_@P-P@WS: Fe_3_O_4_- and polydimethylsiloxane-sprayed polypyrrole-coated polydopamine-modified superhydrophobic wood sponge; C18TMS: octadecyltrimethoxysilane; DA: dopamine; Py: pyrrole; SDBS: sodium dodecylbenzene sulfonate; PDMS: polydimethylsiloxane.

As described in Scheme 2, the in situ polymerization method was employed to sequentially deposit PDA and PPy onto the surface of the WS to obtain P-P@WS. Then, Fe_3_O_4_ particles modified with hydrophobic C18TMS and the low-surface-energy material PDMS were sprayed onto the surface of sponge using a spray gun to prepare Fe_3_O_4_@P-P@WS.

**Figure 5:**
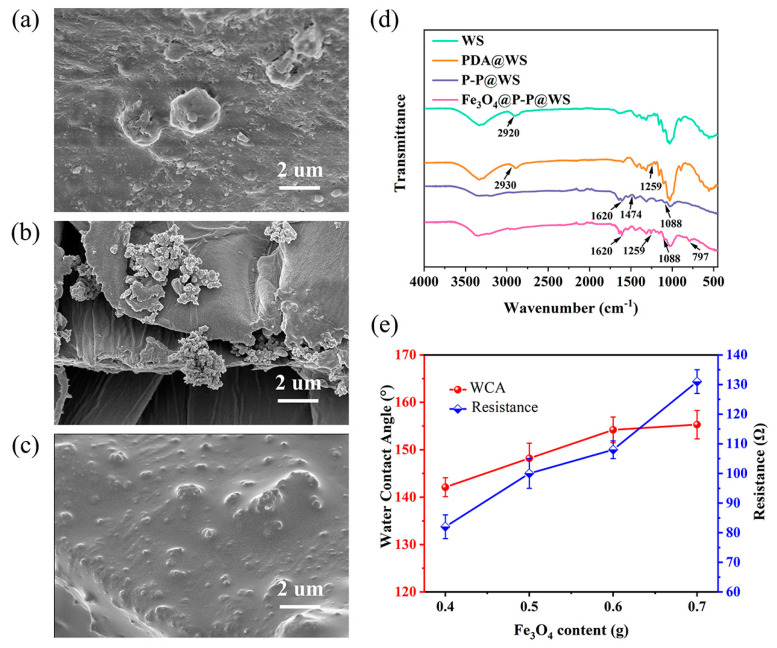
SEM images of (**a**) PDA@WS, (**b**) P-P@WS, and (**c**) Fe_3_O_4_@P-P@WS. (**d**) FTIR spectra of WS, PDA@WS, P-P@WS, and Fe_3_O_4_@P-P@WS. (**e**) Effect of Fe_3_O_4_ content on the WCA and resistance of Fe_3_O_4_@P-P@WS. SEM: scanning electron microscopy; PDA@WS: polydopamine-modified wood sponge; P-P@WS: polypyrrole-coated polydopamine-modified wood sponge; Fe_3_O_4_@P-P@WS: Fe_3_O_4_- and polydimethylsiloxane-sprayed polypyrrole-coated polydopamine-modified superhydrophobic wood sponge; FTIR: Fourier-transform infrared spectroscopy; WCA: water contact angle.

As illustrated in Figure 5a, the WS framework was coated with spherical PDA particles. After the in situ polymerization of PPy, the number of granular structures on the sponge framework increased significantly (Figure 5b), confirming the successful modification of the WS framework by both PDA and PPy. Figure 5c shows the uniformly distributed Fe_3_O_4_ particles on the hydrophobically modified surface, which exhibits a highly reflective appearance due to the PDMS coating. FTIR analysis (Figure 5d) further verified the successful modification of PDA, as indicated by the appearance of characteristic peaks in the PDA@WS infrared spectrum. Notably, a peak at 2930 cm^−1^ corresponding to the C-H stretching vibration on the aromatic ring side chains and a peak at 1259 cm^−1^ representing the stretching vibration of the phenolic hydroxyl group were observed [45]. The infrared spectrum of P-P@WS displayed the characteristic peak of the pyrrole (Py) ring at 1474 cm^−1^, associated with the C-N stretching vibration, alongside the peak at 1620 cm^−1^ representing the C-C and C=C stretching vibrations, present in both P-P@WS and Fe_3_O_4_@P-P@WS spectra [46]. Additionally, the PDMS coating exhibited peaks at 1259 cm^−1^ and 797 cm^−1^ in the Fe_3_O_4_@P-P@WS spectrum, indicating the symmetric stretching and rocking vibrations of Si-CH_3_, respectively [47]. These results confirm the successful surface modification of the sponge with PPy, PDA, PDMS, and Fe_3_O_4_.

To investigate the effect of Fe_3_O_4_ content on the resistance and WCA, the experiment adjusted the Fe_3_O_4_ content in increments of 0.1 g, ranging from 0.4 g to 0.7 g (Figure 5e). When the Fe_3_O_4_ content exceeded 0.6 g, the WCA surpassed 150°, achieving superhydrophobicity. However, a significant change in resistance was observed at 0.7 g Fe_3_O_4_ compared to 0.6 g Fe_3_O_4_, so the Fe_3_O_4_ content was set at 0.6 g for the study.

**Figure 6:**
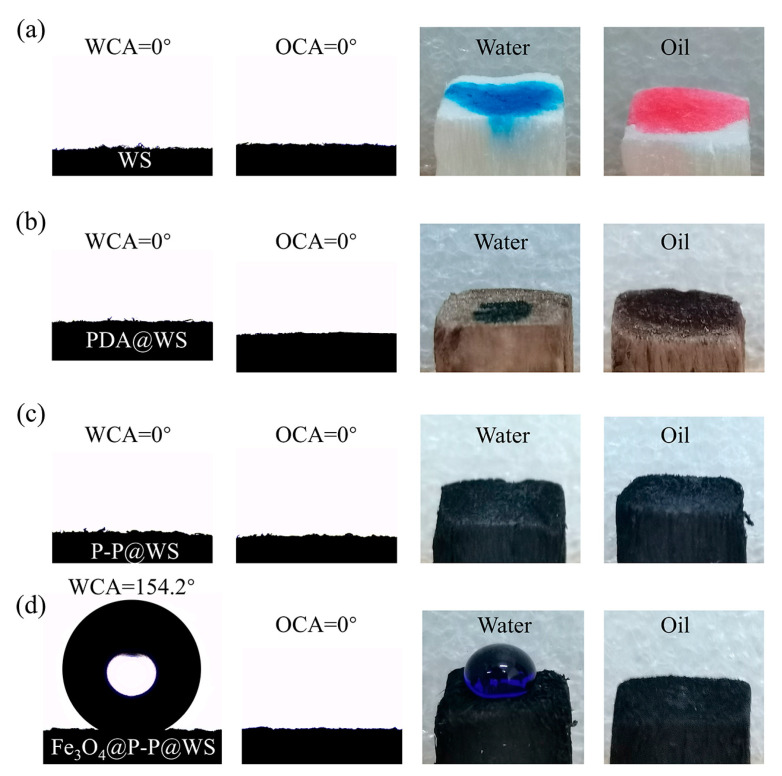
WCA and OCA of (**a**) WS, (**b**) PDA@WS, (**c**) P-P@WS, and (**d**) Fe_3_O_4_@P-P@WS. WCA: water contact angle; OCA: oil contact angle; WS: wood sponge; PDA@WS: polydopamine-modified wood sponge; P-P@WS: polypyrrole-coated polydopamine-modified wood sponge; Fe_3_O_4_@P-P@WS: Fe_3_O_4_- and polydimethylsiloxane-sprayed polypyrrole-coated polydopamine-modified superhydrophobic wood sponge.

The WCA and oil contact angle (OCA) on sponge surfaces were visualised under different modification steps using water and pumped oil (Figure 6). As shown in Figure 6a–c, the surfaces of WS, PDA@WS, and P-P@WS exhibited both hydrophilicity and oleophilicity, with WCA = 0 and OCA = 0. However, the addition of PDMS and Fe_3_O_4_ (Figure 6d) resulted in a superhydrophobic (WCA = 154.2°) and oleophilic (OCA = 0) surface, making oil–water separation possible.

**Figure 7:**
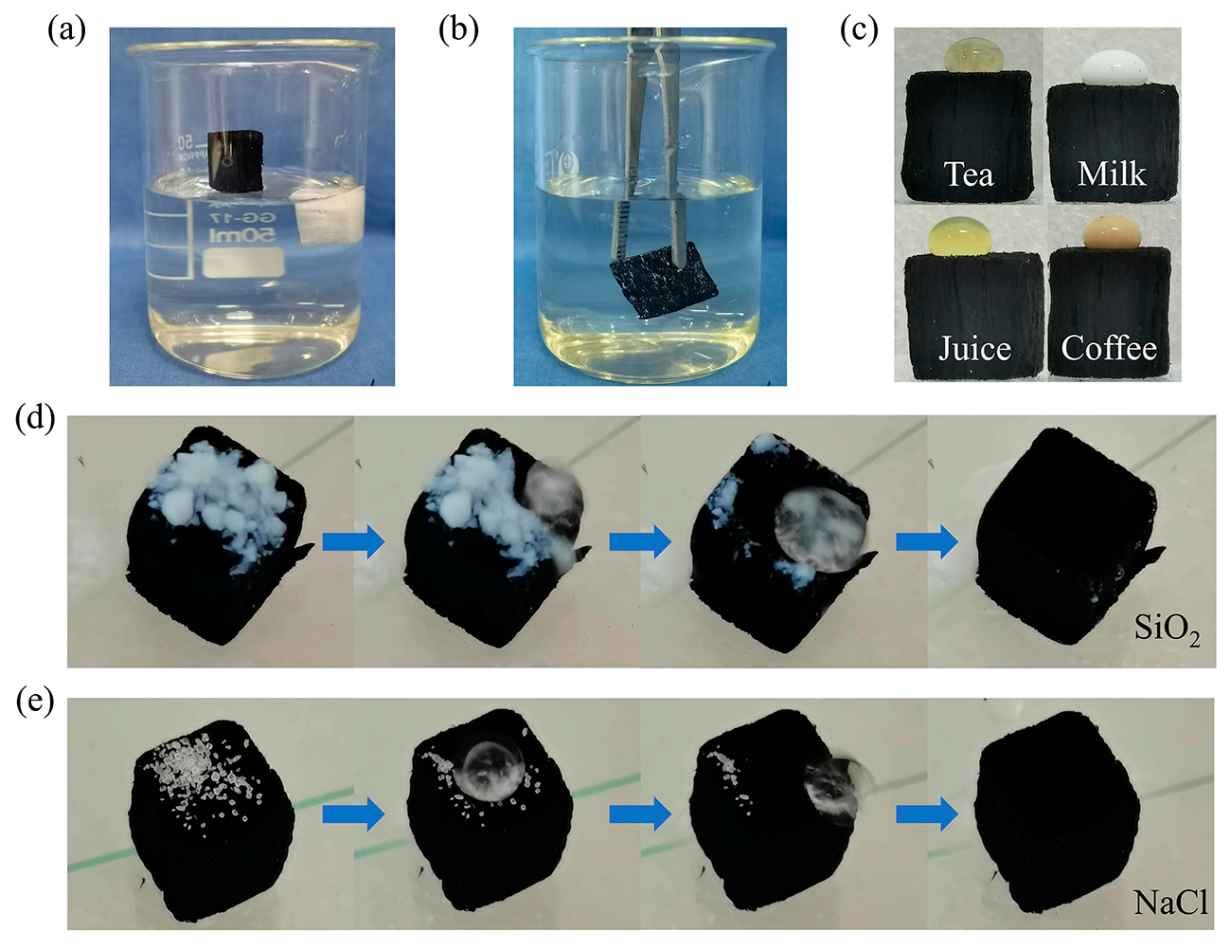
(**a**) WS and Fe_3_O_4_@P-P@WS placed in water simultaneously. (**b**) Fe_3_O_4_@P-P@WS is pressed into the water under external force. (**c**) Wettability of tea, milk, juice, and coffee on the surface of the Fe_3_O_4_@P-P@WS. Self-cleaning process of Fe_3_O_4_@P-P@WS for (**d**) SiO2 and (**e**) NaCl. WS: wood sponge; Fe_3_O_4_@P-P@WS: Fe_3_O_4_- and polydimethylsiloxane-sprayed polypyrrole-coated polydopamine-modified superhydrophobic wood sponge.

When Fe_3_O_4_@P-P@WS and WS were simultaneously introduced into water (Figure 7a), the Fe_3_O_4_@P-P@WS remained buoyant on the surface due to its superhydrophobic properties, whereas the WS sank as it absorbed water. As shown in Figure 7b, when Fe_3_O_4_@P-P@WS was forcibly submerged, a silvery, mirror-like reflection formed around the material, consistent with the Cassie model [48]. Additionally, the modified sponge exhibited exceptional resistance to wetting when exposed to various common liquids such as tea, milk, juice, and coffee (Figure 7c). In real-world applications, environmental particulates like dust may accumulate on the superhydrophobic surface of the sponge, potentially influencing its oil–water separation efficiency.

### 3.4. Self-Heating Performance of Fe_3_O_4_@P-P@WS

Recent studies on PDA have demonstrated that its indole structure and non-covalent interactions contribute to its excellent photothermal properties, converting light energy into heat energy through non-radiative transitions after absorbing solar energy [49,50]. Additionally, similar to mussel foot proteins, the structure of PDA exhibits strong adhesive properties influenced by amino acids, particularly 3,4-dihydroxyphenylalanine (DOPA). The catechol group (3,4-dihydroxy) in DOPA is the key to this adhesion, granting PDA superior adhesive and antioxidant properties [51,52] even in humid environments. Therefore, PDA is an excellent photothermal material and adhesive. In this study, PDA was utilized to impart photothermal properties to the sponge through in situ polymerization, enabling localized heat generation. The adhesive properties of PDA also facilitated the robust attachment of PPy and Fe_3_O_4_ to the skeleton of the WS, enhancing its mechanical performance. However, relying solely on sunlight for heat conversion has limitations. In addition to its photothermal properties, PPy is a good conductive polymer. Under insufficient light conditions, the conductivity of PPy can be utilized to generate Joule heat given an external power source, enabling continuous photothermal conversion throughout the day.

**Figure 8:**
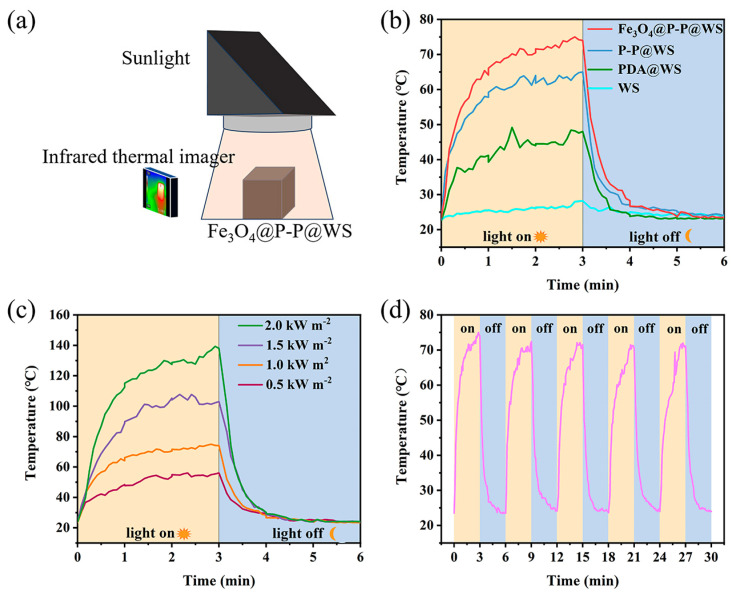
(**a**) Schematic diagram of surface temperature analysis experiment under simulated sunlight. (**b**) Variation in surface temperature of different coatings under a solar intensity of 1 kW m^−2^. (**c**) Variation in surface temperature of Fe_3_O_4_@P-P@WS under different solar intensities. (**d**) Variation in surface temperature of Fe_3_O_4_@P-P@WS during five cycles of light on and off. WS: wood sponge; PDA@WS: polydopamine-modified wood sponge; P-P@WS: polypyrrole-coated polydopamine-modified wood sponge; Fe_3_O_4_@P-P@WS: Fe_3_O_4_- and polydimethylsiloxane-sprayed polypyrrole-coated polydopamine-modified superhydrophobic wood sponge.

The self-heating capability of the samples under simulated sunlight was first analyzed using an infrared thermal imager (Figure 8a). Under a solar intensity of 1.0 kW m^−2^, the photothermal conversion capabilities of different coatings varied significantly (Figure 8b). The surface temperature of the unmodified WS remained unchanged after 3 min of exposure, remaining at room temperature, indicating its lack of photothermal properties. In contrast, the surface temperature of PDA-coated PDA@WS rose to 47.8 °C, confirming the efficient photothermal conversion capability of PDA. The surface temperatures of P-P@PDA and Fe_3_O_4_@P-P@WS increased further to 64.6 °C and 74 °C, respectively, demonstrating the superior photothermal performance of PPy and Fe_3_O_4_. As shown in Figure 8c, as the solar intensity increased from 0.5 kW m^−2^ to 2.0 kW m^−2^, the surface temperature of Fe_3_O_4_@P-P@WS rose from 56 °C to 139.3 °C, reflecting a linear correlation between steady-state temperature and light intensity. The samples quickly reached stable temperatures under varying light intensities, and after the illumination was halted, the surface temperature rapidly returned to room temperature. Moreover, the steady-state temperature of Fe_3_O_4_@P-P@WS remained consistent during repeated light on/off cycles (Figure 8d), indicating stable photothermal conversion performance.

**Figure 9:**
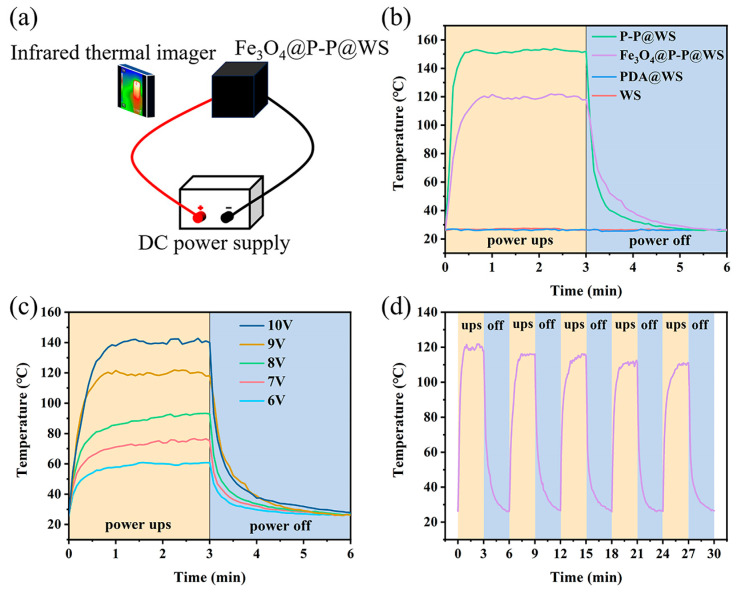
(**a**) Schematic diagram of surface temperature analysis experiments under applied voltage. (**b**) Surface temperature variation of different coatings at 9 V. (**c**) Surface temperature variation of the Fe_3_O_4_@P-P@WS under different voltages. (**d**) Surface temperature variation of the Fe_3_O_4_@P-P@WS during five cycles of power ups-off. Fe_3_O_4_@P-P@WS: Fe_3_O_4_- and polydimethylsiloxane-sprayed polypyrrole-coated polydopamine-modified superhydrophobic wood sponge.

Next, the thermal conversion capability of Fe_3_O_4_@P-P@WS under an applied voltage was analyzed using an infrared thermal imager (Figure 9a). When the output voltage was fixed at 9 V (Figure 9b), the temperatures of the WS and PDA@WS remained unchanged, indicating that neither the original WS nor the PDA@WS possessed electrical conductivity and could not generate Joule heat. After the conductive polymer PPy was added, the surface temperature of the P-P@WS rapidly increased to 152.2 °C under a 9 V voltage. However, after the spray application of PDMS and modified Fe_3_O_4_, the surface steady-state temperature of the Fe_3_O_4_@P-P@WS was 120.5 °C, lower than the 152.2 °C observed on the surface of the P-P@WS. This is because PDMS and modified Fe_3_O_4_ nanoparticles are almost non-conductive, and the increased surface resistance of the sample after coating with the superhydrophobic layer resulted in less heat generation according to the Joule’s law formula $Q=\frac{U^{2}T}{R}$. As shown in Figure 9c, as the voltage increased from 6 V to 10 V, the surface temperature of the Fe_3_O_4_@P-P@WS increased from 60.8 °C to 142.5 °C, indicating a linear relationship between the steady-state temperature and the voltage intensity. The Fe_3_O_4_@P-P@WS maintained stable steady-state temperatures during repeated on/off cycles at a fixed voltage of 9 V (Figure 9d).

**Figure 10:**
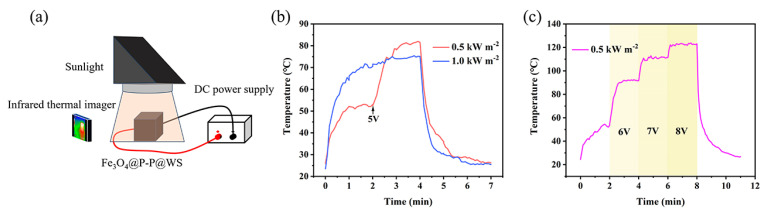
(**a**) Schematic diagram of surface temperature analysis experiments under combined simulated sunlight and voltage. (**b**) Comparison between the surface temperature of the Fe_3_O_4_@P-P@WS under a combination of 0.5 kW m^−2^ sunlight and 5 V voltage with surface temperature under 0.5 kW m^−2^ sunlight alone, (**c**) surface temperature variation of the Fe_3_O_4_@P-P@WS under a combination of 0.5 kW m^−2^ sunlight and different voltage intensities. Fe_3_O_4_@P-P@WS: Fe_3_O_4_- and polydimethylsiloxane-sprayed polypyrrole-coated polydopamine-modified superhydrophobic wood sponge.

Solar intensity may not always reach 1.0 kW m^−2^ across different latitudes, at various times of day, or under unfavorable weather conditions. Therefore, the synergistic effect of sunlight and applied voltage is crucial for achieving effective oil adhesion and absorption under all-weather conditions. The surface temperature variations of Fe_3_O_4_@P-P@WS, under the combined influence of external voltage and simulated sunlight, were analyzed using an infrared thermal imager (Figure 10a). As illustrated in Figure 10b, when the solar intensity was limited to 0.5 kW m^−2^, applying a voltage of 5 V in conjunction with the 0.5 kW m^−2^ sunlight increased the surface steady-state temperature to 85.2 °C, surpassing the 74 °C attained under 1.0 kW m^−2^ sunlight alone. This demonstrates the potential for synergistic enhancement between sunlight and external voltage. Furthermore, Figure 10c shows that the surface temperature of Fe_3_O_4_@P-P@WS increased progressively with rising voltage at a fixed light intensity of 0.5 kW m^−2^. In conclusion, utilizing solar energy as the primary energy source, supplemented by external voltage when sunlight is insufficient, allows Fe_3_O_4_@P-P@WS to efficiently generate heat under diverse environmental conditions. This generated heat can significantly improve the oil adhesion efficiency of Fe_3_O_4_@P-P@WS.

### 3.5. Oil Absorption Properties of Fe_3_O_4_@P-P@WS

**Figure 11:**
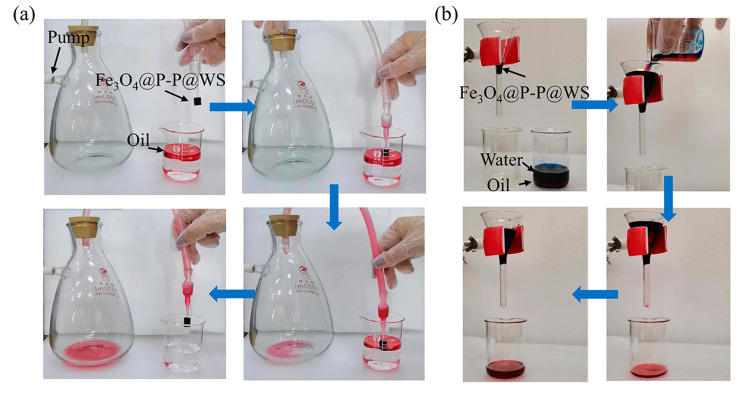
(**a**) Continuous separation of low-density, low-viscosity oils using Fe_3_O_4_@P-P@WS. (**b**) Continuous separation of high-density, low-viscosity oils facilitated by Fe_3_O_4_@P-P@WS. Fe_3_O_4_@P-P@WS: Fe_3_O_4_- and polydimethylsiloxane-sprayed polypyrrole-coated polydopamine-modified superhydrophobic wood sponge.

To verify the suitability of Fe_3_O_4_@P-P@WS for the continuous separation of various oily wastewaters, distinct devices were designed based on the physical properties of different oily substances (Figure 11a). For the separation of oils with a density lower than water and relatively low viscosity, a system consisting of an external vacuum pump, oil tubing, and Fe_3_O_4_@P-P@WS was employed. Owing to the superhydrophobic and superoleophilic nature of Fe_3_O_4_@P-P@WS, toluene dyed with Oil Red O was readily absorbed by the sponge through capillary action. The toluene was continuously drawn into a collection flask by the vacuum pump until all of the floating toluene on the water surface had been removed. The suction force of the vacuum pump was, however, insufficient to overcome the pressure of the air film formed between the superhydrophobic coating and the water. This allowed for the efficient separation of light oils from the water surface without interference from the underlying water.

As depicted in Figure 11b, gravity-driven separation was employed for high-density, low-viscosity oils. Dichloroethane, stained with Oil Red O, permeated the Fe_3_O_4_@P-P@WS and flowed into a lower collection container through capillary action, aided by the force of gravity. Meanwhile, the superhydrophobic coating on the sponge surface effectively repelled MB-dyed water, preventing its passage and facilitating the successful separation of submerged heavy oils from the aqueous phase.

**Figure 12:**
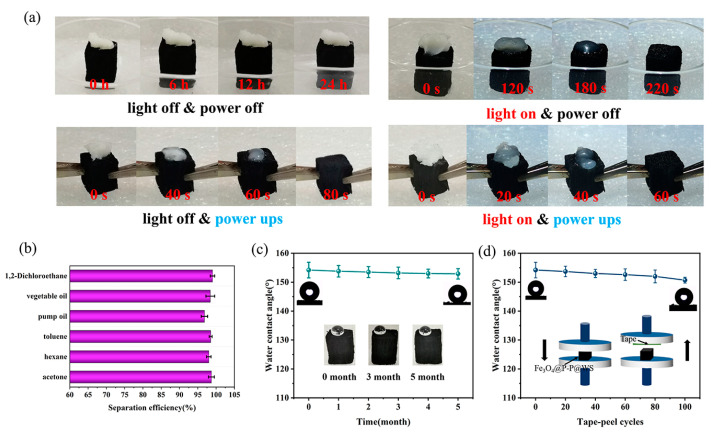
(**a**) Adsorption of high-viscosity oil by Fe_3_O_4_@P-P@WS. (**b**) Separation efficiency of Fe_3_O_4_@P-P@WS for different oils. (**c**) Variation curve of WCA on Fe_3_O_4_@P-P@WS surface over time. (**d**) Variation curve of WCA on Fe_3_O_4_@P-P@WS surface after 100 tape peeling tests.

Solar- and electric-driven techniques were employed for the separation of high-viscosity oils (Figure 12a). In the absence of light or electrical input, the viscous oil on the surface of the Fe_3_O_4_@P-P@WS remained unadsorbed, showing no significant change even after 24 h. However, under sunlight with an intensity of 1.0 kW m^−2^, the oil was fully adsorbed within 220 s, markedly reducing the adsorption time. When a 9 V voltage was applied, the adsorption time decreased further to just 80 s, as the steady-state surface temperature of the sponge reached 120.5 °C, compared to 7 °C under sunlight alone (Figure 8b and Figure 9b). This demonstrates that oil viscosity decreases as temperature increases. Under the combined action of sunlight (1.0 kW m^−2^) and voltage (9 V), the complete adsorption of viscous oil occurred within only 60 s.

The separation efficiency of the Fe_3_O_4_@P-P@WS for different oils was evaluated using a separation efficiency formula (Equation (1)). Acetone, n-hexane, toluene, pump oil, and dichloroethane were employed as the oil phases. As shown in Figure 12b, the sponge achieved a separation efficiency of over 96.5% for all six oils, with the highest efficiency of 98.9% achieved for dichloroethane. In addition, continuous oil–water separation experiments using 1,2-dichloroethane were carried out for thirty repetitions. According to the experimental results, after thirty repeated separations, the separation efficiency of Fe_3_O_4_@P-P@WS for 1,2-dichloroethane decreased from the initial 98.9% to 97%. These findings demonstrated that the Fe_3_O_4_@P-P@WS fabricated in this research was highly efficient in adsorbing oils with different viscosities and densities, rendering it a prospective material for oil–water separation.

To verify the stability and robustness of the superhydrophobic performance of the Fe_3_O_4_@P-P@WS, the prepared superhydrophobic sponge was tested to determine its WCA after being exposed to air for different durations (Figure 12c). After five months, the WCA decreased only slightly from the initial 154.2° to 152.9°, indicating that it still maintained its superhydrophobic properties. Furthermore, to additionally illustrate the mechanical durability of the superhydrophobic coating, a tape peeling test was carried out with 3M600 tape and a universal testing machine (Figure 12d). After 100 peeling tests, the WCA dropped to 150.7°. The experimental outcomes suggest that the superhydrophobic coating fabricated in this study shows outstanding mechanical performance and stability.

In addition, a comparison was made between Fe_3_O_4_@P-P@WS and the superhydrophobic sponges prepared from commercial sponges that are currently widely used, such as polyurethane sponge (PU) and melamine sponge (MS). The results are shown in Table 1. It can be seen from the table that the superhydrophobic sponge obtained in this study can be comparable to the superhydrophobic sponges prepared from commercial sponges in terms of water contact angle (WCA) and oil–water separation ability. Therefore, it can be concluded that the superhydrophobic wood sponge prepared in this experiment, which can adsorb viscous oil, has broad application prospects.

## 4. Conclusions

In this study, an environmentally friendly three-dimensional wood sponge (WS) with all-weather oil adhesion capability was developed. The fabrication process involved removing lignin and hemicellulose from natural wood (NW) through a two-step chemical treatment, followed by freeze-drying to create a three-dimensional cellulose-based sponge structure. Subsequent in situ polymerization attached the photothermal conversion material polydopamine (PDA) and conductive polymer polypyrrole (PPy) onto the sponge framework, with hydrophobic modification applied via a spray coating technique. The WS, produced through this two-step method, exhibited excellent elasticity and successfully addressed the issue of secondary pollution during both production and recycling. The Fe_3_O_4_@P-P@WS demonstrated outstanding superhydrophobicity and superoleophilicity, enabling continuous separation of low-density, low-viscosity oils and high-density, low-viscosity oils through the action of either an external vacuum pump or gravity. Furthermore, the modified sponge possessed impressive self-cleaning properties and durability, ensuring stable oil–water separation performance in practical applications.

Moreover, the superhydrophobic sponge possessed excellent photothermal conversion and Joule heating capabilities, allowing it to generate substantial heat under sunlight and external voltage. This reduced the oil viscosity, consequently decreasing the oil adhesion time. Compared to other oil–water separation sponges, this study utilized natural, renewable wood as the raw material, effectively solving the problem of secondary pollution while also enabling the sponge to generate its own heat using both solar and electrical energy. These features not only conserve energy but also facilitate all-weather oil adhesion. Furthermore, the preparation method employed in this experiment was simple and cost-effective, making the synthesized sponge highly promising for applications in the treatment of oily wastewater and oil adhesion.

## Figures and Tables

**Table 1:** WCA and oil–water separation efficiency of different superhydrophobic sponges.

Sponge Type	WCA	Oil Water Separation Efficiency	References
PU	151.32°	97.14–99.15%	[20]
PU	156.2°	>94%	[53]
MS	150.5°	99%	[15]
MS	155.4°	98.52%	[16]

## Data Availability

The original contributions presented in the study are included in the article, further inquiries can be directed to the corresponding author.
